# Expressed Sequence Tags from Cephalic Chemosensory Organs of the Northern Walnut Husk Fly, *Rhagoletis suavis*, Including a Putative Canonical Odorant Receptor

**DOI:** 10.1673/031.010.5101

**Published:** 2010-06-03

**Authors:** Karlene M. M. Ramsdell, Sheila A. Lyons-Sobaski, Hugh M. Robertson, Kimberly K. O. Walden, Jeffrey L. Feder, Kevin Wanner, Stewart H. Berlocher

**Affiliations:** ^1^ Department of Entomology, 320 Morrill Hall, 505 S. Goodwin Ave., University of Illinois at Urbana-Champaign, Urbana, IL 61801; ^2^ Department of Biology, Albion College, 611 E. Porter St., Albion, Michigan 49224; ^3^ Department of Biological Sciences, P.O. Box 369, Galvin Life Science Center, University of Notre Dame, Notre Dame, IN 46556-0369.

**Keywords:** host race, *jugions nigra*, olfaction, odorant receptor, *Rhagoletis*, Tephritidae, speciation

## Abstract

*Rhagoletis* fruit flies are important both as major agricultural pests and as model organisms for the study of adaptation to new host plants and host race formation. Response to fruit odor plays a critical role in such adaptation. To better understand olfaction in *Rhagoletis*, an expressed sequence tag (EST) study was carried out on the antennae and maxillary palps of *Rhagoletis suavis* (Loew) (Diptera: Tephritidae), a common pest of walnuts in eastern United States. After cDNA cloning and sequencing, 544 ESTs were annotated. Of these, 66% had an open reading frame and could be matched to a previously sequenced gene. Based on BLAST sequence homology, 9% (49 of 544 sequences) were nuclear genes potentially involved in olfaction. The most significant finding is a putative odorant receptor (OR), *RSOr1*, that is homologous to *Drosophila melanogaster Or49a* and *Or85f*. This is the first tephritid OR discovered that might recognize a specific odorant. Other olfactory genes recovered included odorant binding proteins, chemosensory proteins, and putative odorant degrading enzymes.

## Introduction

Species of the genus *Rhagoletis* are important pests of fruits such as apples, cherries, tomatoes, walnuts, and blueberries. They are equally important as the focus of the debate about the possibility of sympatric speciation via the formation of host races on new host plants (Bush 1966; Berlocher and Feder 2002). In the case of the apple host race of *Rhagoletis pomonella*, two key adaptations arose approximately 150 years ago in the ancestral (and still extant) hawthorn race that allowed colonization of apple. One is alteration of the olfactory response so that both sexes are attracted to the odor of the new host apple (Linn et al. 2003, 2004; Dambroski et al. 2005), and the other is shifting life history phenology to match the fruit ripening time of apple (Filchack et al. 2000). This study is the first attempt to catalog genes involved in olfaction in *Rhagoletis* by carrying out an expressed sequence tag (EST) project on the antennae and maxillary palps of *Rhagoletis suavis* (Loew) (Diptera: Tephritidae). This species was used because it can be obtained more easily in the large numbers required for an EST project on olfactory organs than *R. pomonella* can.

Many features of the molecular biology of olfaction in *Rhagoletis* can be anticipated from what is known of olfaction in *Drosophila melanogaster*, which is a key model organism for studying olfaction (Rützler and Zwiebel 2005; Hallem et al. 2006; Vosshall and Stocker 2007). Two major gene families involved are the odorant binding proteins (OBPs) (Hekmat-Scafe et al. 2002) and the odorant receptors (ORs) (Robertson et al. 2003). OBPs are usually highly expressed, which makes detection in antennal EST projects likely (e.g. Robertson et al. 1999); whereas ORs are generally expressed at such low levels that they are difficult to obtain with this method. Based on the *D. melanogaster* genome, it was anticipated that the most important recoveries from this EST project would be key olfactory gene products such as ORs and OBPs. However, other classes of genes have been proposed as having a possible role in olfaction, such as chemosensory proteins (Briand et al. 2002; Laitigue et al. 2002) and odorant degrading enzymes, as well as genes that are of general interest.

## Materials and Methods

### Flies and collection of antennae and palps

Collection of large numbers of *Rhagoletis* flies is most easily accomplished by rearing larvae from infested fruit (*Rhagoletis* life history is described by Boller and Prokopy 1975). In the fall of 2000, approximately 50,000 *R. suavis* (Loew) larvae were reared from black walnut, *Juglans nigra* L. (Fagales: Juglandaceae), fruit from sites near White Heath, Illinois (Piatt County). Pupae were placed in a 4° C cold room to break diapause and then removed in batches throughout the spring of 2001. Emerging flies were placed in cages with food and water (Prokopy and Bush 1973) until they could be processed. Processing was carried out as rapidly as possible after eclosion because young adults were assumed to have the highest expression of olfactory receptors. Heads from live flies were removed and accumulated at -80° C. The day before RNA extraction, the frozen heads were shaken on a soil sieve to harvest antennae. Maxillary palps and major head bristles that may also have chemoreceptors were harvested incidentally. Maxillary palps have sensilla used in odor recognition and express gustatory receptors, but mRNA would not have been obtained from major bristles because the cell bodies are not in the bristles. The shaking and sieving was not severe enough to break the heads, so there was no contamination from brain or eye tissues.

### RNA extraction and cDNA library construction

Total RNA was isolated from antennae and maxillary palps using a guanidinium thiocyanate/phenol-chloroform extraction protocol (RNA Isolation Kit, Stratagene, www.stratagene.com). mRNA was purified from total RNA using a Poly(A) Quik® mRNA Isolation Kit (Stratagene, www.stratagene.com), which utilizes an oligodT cellulose column. A unidirectional plasmid cytomegalovirus-polymerase chain reaction cDNA library primed with oligo-dT was constructed by Stratagene using PCR amplification. The plasmid library was transformed into Stratagene's host strain Epicurian Coli® XL-IO Gold™. For further details of molecular methods and results see Ramsdell (2004).

### Clone sampling & DNA sequencing

Plasmid clones were sampled by plating the library onto LB-kanamycin agar and picking colonies. Colonies were individually transferred to 96-well plates. Each well contained 80 µl of a 30% (v/v) glycerol-LB mixture. Six plates were prepared and submitted to the W.M. Keck Center for Comparative and Functional Genomics (University of Illinois at Urbana-Champaign) for sequencing from the 5′ end using ABI automation. Clones of interest were cultured and purified, and the insert was sequenced from both directions when necessary.

### Sequence analysis

Sequences were edited with Microsoft Excel® and BBEdit Lite (Bare Bones Software, Inc., www.barebones.com). DNA Strider v1.1 (Marck 1988) was used for protein translations, to find open reading frames, convert reverse complement sequence reads, and generate Kyte-Doolittle hydropathy plots (Kyte and Doolittle 1982). BLAST (Altschul et al., 1990, 1997) was used with networked servers (National Center for Biotechnology Information; http://www.ncbi.nlm.nih.gov) to find the most similar sequence matches to the *R. suavis* ESTs in the GenBank databases. Significantly similar matches had E values of 10^-4^. An initial screen using the tblastn option (translated DNA query searching translated DNA database) was followed with both nucleotide and protein BLAST searches (blastn, blastx, blastp) of the largest open reading frames. Searches were generally restricted to Diptera. Sequences of interest were aligned using Clustal X 2.0 (Larkin et al. 2007) using default settings. EST sequences were deposited in the dbEST EST database at the National Center for Biotechnology Information (Accessions EX453814 EX454354).

To show the relationships of the *Drosophila melanogaster* OR sequences that are most similar to the *R. suavis* OR, a neighbor-joining tree of corrected distances was built using Clustal X (Larkin et al. 2007). Bootstrapping was performed with Clustal X with 10,000 pseudoreplications.

## Results

### Recovery of ESTs

A total of 544 clones was sequenced, with an average length 532.02 ± SE 9.88 bp (range 14 to 967 bb). A wide variety of gene transcripts was obtained. As expected from a normalized library, 418 (76.8%) of the sequences were unique. The largest number of duplicates was 18 for a sequence similar to DmCG13095 (a peptidase). Of the 544 total sequences, 186 had no obvious ORF and did not produce a significant BLASTx match with a known protein sequence in GenBank. Of the 358 sequences with an ORF, 86 produced either a weak match with a known gene, or a low E-value match with a sequence of unknown function. Of the 313 sequences with a significant match to a sequence with a known function, 37 were mitochondrial and 276 were nuclear. As expected, protein BLAST searches yielded much smaller E values than did nucleotide searches. The exceptions involved nucleotide matches with sequences from other tephritid flies (*R. pomonella* and the medfly *Ceratitis capitata*), which usually resulted in the smallest E values, presumably because 3′ UTRs retained some sequence similarity.

A representative set of the nuclear matches is shown in 3 1. Given that mRNA was extracted from antennae and maxillary palps, it is not surprising that 48 (9%) of the sequences had a function or putative function relating to chemoreception. Also, 24 of the sequences had a known or possible role in development. This finding is not surprising because the source flies were young adults that were not fully mature. Also included in Table 1 are a few sequences that have been implicated in diapause and life history; such genes were not the target of this study, but they are noted because diapause is critical to host race formation in *Rhagoletis*. Although they do not appear to play a role in diapause initiation, heat shock loci can be up-regulated during diapause (Rinehart et al. 2007).

### Chemosensory proteins

Thirteen sequences were recovered that coded for two different chemosensory proteins (CSPs), RsCSP1 and CSP2. The *R. suavis* CSPs matched only chemosensory proteins in the public databases and were identified as belonging to the conserved domain of the CSP family. Proteins from *D. melanogaster* Antennal Protein 10 (A10 or OS-D) and Ejaculatory Bulb Protein III (PEBme III), were the best matches for RsCSP1 and RsCSP2, respectively. A10 and RsCSP1 had a pairwise amino acid identity of 66%, and RsCSP2 and PEBme III were 82% identical. The *R. suavis* CSPs have an amino acid identity of 45.7%; the mature forms are 50.9% identical. RsCSP1 was 155 amino acids in length, including a signal peptide of 21 amino acids, and RsCSP2 had a length of 127 with its 18 amino acid signal.

### Odorant binding proteins

Nine OBPs, RsObp1 to RsObp9, were recovered. All had top matches to dipteran OBPs in the public databases. The Kyte-Doolittle hydropathy plots of the nine proteins showed typical OBP profiles with hydrophobic peptide signals (Peng and Leal 2001). Including their peptide signals, the OBPs ranged in length from 124 to 164 amino acids. Overall, the *R. suavis* OBPs were diverse and showed little conservation of amino acid residues. The mature OBPs had mean pairwise amino acid identities of 19.9%, with a range of 7.4 to 55.9%. Signal peptides were 15 to 26 amino acids in length (Ramsdell 2004).

### Odorant receptor protein

**Figure 1.**
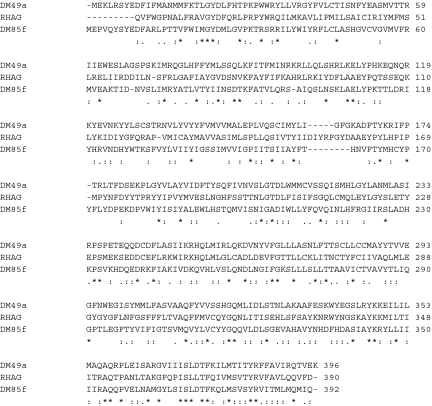
Clustal X alignment of the *Rhagoletis suavis* receptor RsOr1, and *Drosophila melanogaster Or49a* and 85f, which are the most similar sequences to RsOr1. Symbols: ^*^ = identical amino acids, : = conservative substitutions, . = semiconservative substitutions. High quality figures are available online.

**Figure 2.**
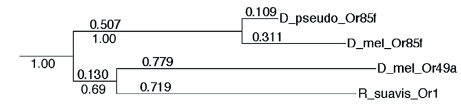
Neighbor-joining tree of sequences similar to the *Rhagoletis suavis* odorant receptor (*Drosophila pseuodooscura* Or85f, and *D. melanogaster* Or85f and Or49a). Distances are percent dissimilarities corrected for multiple replacements. Values under the distances are bootstrap values. High quality figures are available online.

**Figure 3.**
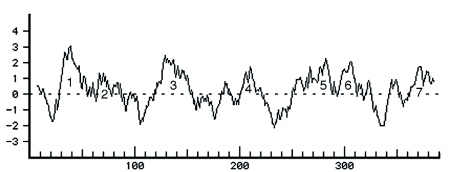
Kyte-Doolittle hydropathy plot for RsOrl. High quality figures are available online.

The *R. suavis* OR sequence (EX453813, 634 bp) was identified as an OR because a protein BLAST search of a 450 bp/150 amino acid ORF significantly (2E-04) matched *DmOr49a*. Resequencing of the clone from both ends revealed an unambiguous match with two *D. melanogaster* OR sequences. These were *DmOr49a* (4E-56, amino acid identity = 31%) and *DmOr85f* (1E-37, amino acid identity = 26%). The alignment of these three sequences is shown in Figure 1. Based on the alignment, it is likely that a few amino acids were missing at the N-terminus of the *R. suavis* sequence. To increase the likelihood that the nearest known homolog of the *R. suavis* receptor was found, the nine *Drosophila* OR sequences were included in the neighbor-joining tree analysis, ranked in order of decreasing E value, between *DmOr49a* and an *Anopheles gambiae* receptor (AGAP001912, 8E-28). The resulting neighbor-joining tree (Figure 2, shows only the relevant part of tree, including Or85f from *Drosophila pseuodoobscura* supports the conclusion that the *D. melanogaster* homolog of the *R. suavis* odorant receptor sequence, henceforth RsOr1, was *DmOr49a*. The RsOr1 sequence clearly showed the characteristic hydropathy plot of a 7-transmembrane protein, with alternating hydrophobic and hydrophilic regions (Figure 3).

**Table 1.**
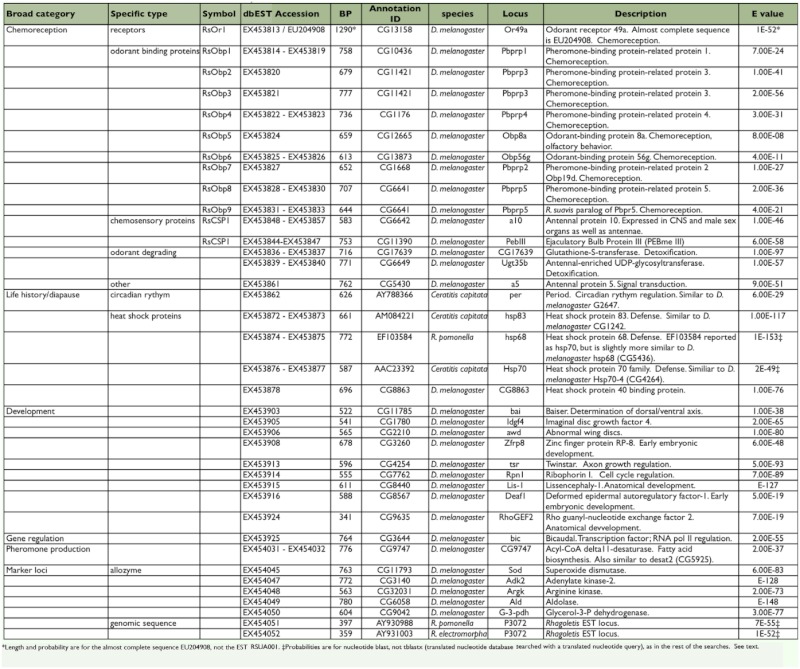
Genes of interest recovered in *R. suavis* antennal EST study.

## Discussion

### Chemosensory Proteins

The function of CSPs is not clear at this point. They are highly expressed in insect antennae, and some work supports a role as olfactory ligand transporters (Briand et al. 2002; Lartigue et al. 2002). Recent work in *Bombyx mori*, however, indicates that they are commonly expressed in many parts of the body in addition to antennae (Gonga et al. 2007). The fact that two different CSPs were recovered in this small study of 544 ESTs indicates that, consistent with other work, CSPs are highly expressed in antennae, but their possible role in *Rhagoletis* olfaction remains uncertain.

### Odorant binding proteins

*Drosophila melanogaster* has 51 OBPs (Hallem et al. 2006, Hallem and Carlson 2006). Thus the recovery of nine different *R. suavis* OBP sequences, all with *D. melanogaster* orthologues, (Table 1) from only 544 ESTs suggests that most, if not all, of the *R. suavis* OBPs could be recovered by a modestly more extensive EST study. The exact role that OBPs could play in host specificity remains unknown; however, it is quite likely that they play a significant part. Recent work on *Drosophila* pheromone reception demonstrates both that OBPs are necessary for chemoreception and that some are highly specific for particular odorants (Xu et al. 2005).

The *R. suavis* OBPs have a mean pairwise amino acid identity of about 20%, which is typical for phylogenetically distant members of the OBP gene family (Robertson et al. 1999). Their diversity, coupled with their apparent homology to *D. melanogaster* OBPs, make them good candidates for use as genetic tools in studies of acalypteran and other dipteran lineages.

### The odorant receptor sequence

Odorant receptors are believed to play a critical role in the host finding behavior in insects, yet they are difficult to obtain without completely sequenced genomes. Only a few odorant receptor sequences have been discovered in insect EST projects (and none of these are published), suggesting a low rate of expression. Indeed Vosshall et al. (1999) noted that *D. melanogaster* ORs were present in fewer than 1 in 500,000 clones in an antennal library. This is lower than this rate of 1 OR in 544 clones, but it is likely that a *Rhagoletis* genome will be necessary to obtain a complete set of OR genes.

*RsOr1* is significant as the first reported putative ligand-binding receptor from a tephritid fly. It is not the first tephritid receptor; that distinction belongs to a receptor recovered from *C. capitata* by Larrson et al. (2004). However, the *C. capitata* OR was homologous to the atypical, “non-canonical” *Or83b*, which plays a role in localizing conventional or “canonical” receptors to the membrane and is highly conserved across insects (Jones et al. 2005). But *Or83b* does not bind odorant ligands, and, thus, its homologs are unlikely to play a direct part in host plant adaptation. *RsOr1*, on the other hand, was clearly homologous to the canonical *DmOr49a*. Unfortunately, it is not possible, at this point, to speculate on the volatile, or volatiles, which elicits a response from *RsOr1*, as the ligands of *DmOr49a* have not yet been determined (Hallem and Carlson 2006).

However, it is probable that the OR sequences, or their expression patterns, or both, differ substantially between *R. suavis* and the apple maggot *R. pomonella*. The fruit volatiles of apples are characterized by high concentrations of esters (Linn et al. 2003; Souleyre et al. 2005), while those of the ancestral host of *R. pomonella*, hawthorns, are characterized by ethyl acetate, long-chain alcohols, and various aldehydes (Linn et al. 2003). But a completely different spectrum of volatiles, dominated by terpenes and terpenoids, occurs in walnut fruits (Hennemanm et al. 2002). Moreover, many of the walnut terpenoids, such as β-pinene, limonene, β-caryophylene, and α-humulene (Hennemanm et al. 2002) did not elicit any responses from the (incomplete) set of ORs tested by Hallem and Carlson (2006). Thus *R. suavis* may provide insights into insect olfaction that are not possible with *Drosophila*.

*R. suavis* may be a good species with which to study the various roles of odorant degrading enzymes in olfaction. As pointed out by Rützler and Zwiebel (2005), odorant degrading enzymes are necessary to remove the signaling molecule after a cell response has been initiated and also because chemosensory systems must be open to the environment. Odorant degrading enzymes may have a secondary role of degrading toxic odorants before they can cause cellular damage. One of the major components of walnut fruit odor is limonene, which is used as an insecticide, and also causes “spontaneous stimulation of sensory nerves” (Weinzierl 1998, p. 106; mechanism not known). Detoxification of limonene in the cutworm *Spodoptera* is reported to be similar to mammalian detoxification (Miyazawa et al. 1998), where oxidative degradation by cytochrome P450s appears to be the most important pathway (e.g., Miyazawa et al. 2002). No cyt P450 sequences were recovered in this study, but they represent one of several pathways that should be studied in olfaction in phytophagous insects.

While tremendous strides have been made in understanding the molecular biology of chemosensation in recent years (Rützler M and, Zwiebel LJ. 2005, Hallem et al. 2006, Vosshall and Stocker 2007), we are still very far from being able to understand the relative importance for host adaptation of peripheral vs. central processes, sequence vs. expression differences, or even the relative importance of the different classes of genes involved. Koop et al. (2008) have recently demonstrated that expression differences for both Ors ORs and OBPs have been involved in the adaptation of *Drosophila sechellia* to its food plant *Morinda citrifolia*. But more classes of molecules will need to be included in future such studies. ODEs and CSPs will certainly need to be added. But even genes that seemingly have little to do with olfaction may be important. For example, Hsp70 genes could affect receptor function in chemosensory cells because of their role of in guiding the folding of proteins (Bukau et al. 2006).

## Abbreviations

CSP: chemosensory protein;
EST: expressed sequence tag;
OBP: odorant binding protein;
OR: odorant receptor
